# Elevated Epidermal Growth Factor (EGF) as Candidate Biomarker of Mood Disorders—Longitudinal Study in Adolescent and Young Adult Patients

**DOI:** 10.3390/jcm10184064

**Published:** 2021-09-08

**Authors:** Maria Skibinska, Pawel Kapelski, Monika Dmitrzak-Weglarz, Natalia Lepczynska, Joanna Pawlak, Joanna Twarowska-Hauser, Aleksandra Szczepankiewicz, Aleksandra Rajewska-Rager

**Affiliations:** 1 Department of Psychiatric Genetics, Poznan University of Medical Sciences, Rokietnicka 8, 60-806 Poznan, Poland; pkapelski@ump.edu.pl (P.K.); mweglarz@ump.edu.pl (M.D.-W.); jopawlak@ump.edu.pl (J.P.); jhauser@ump.edu.pl (J.T.-H.); rajrager@ump.edu.pl (A.R.-R.); 2 Department of Child and Adolescent Psychiatry, Karol Jonscher Clinical Hospital, Poznan University of Medical Sciences, Szpitalna 27/33 St., 60-572 Poznan, Poland; natalia.lepczynska@gmail.com; 3 Laboratory of Molecular and Cell Biology, Department of Pediatric Pulmonology, Allergy and Clinical Immunology, Poznan University of Medical Sciences, Szpitalna 27/33 St., 60-572 Poznan, Poland; alszczep@ump.edu.pl

**Keywords:** adolescent mood disorders, major depressive disorder (MDD), bipolar disorder (BD), brain-derived neurotrophic factor (BDNF), epidermal growth factor (EGF), migration inhibitory factor (MIF), stem cell factor (SCF)

## Abstract

Bipolar disorder (BD) is a chronic mental disorder that affects more than 1% of the population worldwide. Over 65% of patients experience early onset of the disease. Most cases of juvenile bipolar disorder begin with a depressed mood episode, and up to 50% of youth initially diagnosed with major depression go onto developing a BD. Our study aimed to find biomarkers of diagnosis conversion in young patients with mood disorders. We performed a two-year follow-up study on 79 adolescent patients diagnosed with MDD or BD, with a detailed clinical assessment at five visits. We monitored diagnosis change from MDD to BD. The control group consisted of 31 healthy youths. According to the neurodevelopmental and neuroimmunological hypotheses of mood disorders, we analyzed serum levels of brain-derived neurotrophic factor (BDNF), proBDNF, epidermal growth factor (EGF), migration inhibitory factor (MIF), stem cell factor (SCF), and correlations with clinical factors. We detected a significant disease-dependent increase in EGF level in MDD and BP patients at baseline exacerbation of depressive or hypomanic/manic episodes as well as in euthymic state compared to healthy controls. No potential biological predictors of disease conversion were found. Replication studies on a larger cohort of patients are needed.

## 1. Introduction

The lifetime prevalence of mood disorders is about 22% [1]. Providing adequate differential diagnosis between major depressive disorder (MDD) and bipolar disorder (BD) remains a clinical challenge. Misdiagnosis of BD may imply various adverse outcomes, such as inadequate medication, a greater number of relapses and hospitalizations, more lengthy episodes, and a higher level of social impairment [2]. BD is a chronic mental disorder that affects more than 1% of the population worldwide, with onset usually during adolescence. Its course is associated with high morbidity and mortality rates, making bipolar disorder one of the leading causes of disability. Valid biomarkers or diagnostic tools to help clinicians identify individuals at high risk of conversion to bipolar disorder are still lacking [3].

Over 65% of BD patients experience early (before 18 years old) onset of the disease. Early-onset BD is associated with a more severe disease course, psychotic features, mixed episodes, neuropsychological dysfunctions, frequent comorbidity of panic disorder, and drug or alcohol abuse, as well as poorer lithium response and increased risk of suicide [4]. Adolescents with BD have a more prolonged early course and may be less responsive to treatment, characterized by a low symptom remission rate and hypomania/mania recurrence [5]. Affective and behavioral dysregulation, aggressiveness, and irritability are also the features of adolescent BD [5,6]. Age at illness onset might represent a clinical marker useful in defining more homogeneous subgroups in BD. Most cases of early-onset BD begin with a depressed mood episode, and up to 50% of youth initially diagnosed with MDD go on developing a bipolar spectrum disorder [7]. Administering the proper treatment early may result in a better prognosis for youth with BD [8]. Therefore, the validation of early intervention strategies may help improve the illness’s outcome [9].

Neurodevelopmental and neuroinflammatory factors have been implicated in the neurobiology of mental disorders, including BD [10,11]. Evidence linking schizophrenia to neurodevelopmental disturbances is more unequivocal than those for BD. A recent comprehensive review by Kloiber et al. (2020) clearly shows that patients with early-onset BD and psychotic features have more consistent evidence supporting the role of neurodevelopmental pathways. Long-lasting consequences of neurodevelopmental aberrations include immune dysregulation, disruption to the hypothalamic–pituitary–adrenal (HPA) axis, and impairment in oxidative and nitrosative stress pathways [10]. During adolescence, the brain undergoes significant structural changes, with the continued growth of subcortical regions, including the amygdala and thinning of the cortex. Maturation of subcortical–cortical connections occurs [12]. Excessive neuronal pruning and apoptosis during brain maturation might be responsible for the onset of BD, and subsequent impaired neuroplasticity and cellular resilience are liable for further disease progression [13].

Studies on peripheral diagnostic and prognostic biomarkers of mental illnesses are widely conducted, including proteins involved in neurodevelopement and neuroinflammation. Brain-derived neurotrophic factor (BDNF) belongs to the most extensively studied trophic factors in neuropsychiatric disorders. The BDNF protein is widely expressed throughout the developing and adult human brain and is a crucial regulator of neural circuit stabilization and function. It is essential in the regulation of growth, differentiation, and survival of neurons in the brain. BDNF controls the development of dopaminergic, serotonergic, GABA-ergic, and glutamatergic neurons and significantly affects neurogenesis and neuroplasticity [14]. BDNF and its precursor proBDNF exert an opposite effect on synaptic structure, plasticity, transmission, and behavior. BDNF has a neurotrophic and neuroprotective effect, while proBDNF has proapoptotic properties. Furthermore, the processing of proBDNF by intracellular and extracellular proteases is a crucial determinant for neurotrophin function [15].

Epidermal growth factor (EGF) stimulates cell proliferation, growth, and differentiation by binding to its receptor, EGFR. EGF and its receptor expression are detected in the central nervous system in neuronal and glial cells during neurogenesis and in the adult brain [16,17].

Migration inhibitory factor (MIF) is a pleiotropic protein exerting biological features of cytokine and hormone. Studies indicate a proinflammatory role of MIF in immunoinflammatory and autoimmune diseases. The complex role of MIF in the polarization of immune responses, which includes activation of both pro and anti-inflammatory agents, is suggested. MIF may stimulate the secretion of Th1/17 as well as Th2 cytokines. MIF is released by the anterior pituitary and adrenal gland upon hypothalamic–pituitary–adrenal axis (HPA) activation [18,19].

Stem cell factor (SCF, KITLG) plays an essential role in the proliferation and migration of neural progenitor cells. SCF synthesis was detected in neuronal cells in the cerebral cortex, thalamus, cerebellum, and hippocampus. Its receptor C-kit is expressed by neurons and glial cells, including astrocytes and microglia, indicating that SCF signaling is also involved in neuron–glia interactions [20].

### Hypotheses

Studies on biological biomarkers of diagnosis conversion in adolescents with mood disorders in longitudinal design are scarce. According to the neurodevelopmental and neuroimmunological mechanisms underlying the pathogenesis of bipolar disorder, we hypothesize:

(1) There are differences in baseline serum BDNF, proBDNF, EGF, MIF, and SCF levels between depressed, bipolar disorder (in hypomania/mania, or mixed episodes) patients, and controls. (2) Baseline BDNF, proBDNF, EGF, MIF, and SCF levels may predict diagnosis change from unipolar to bipolar disorder in young patients. (3) Baseline protein concentrations correlate to clinical factors (medication status, family history of affective disorders, severity of depressive and manic symptoms, or gender). (4) There are differences between baseline and euthymic state BDNF, proBDNF, EGF, MIF, and SCF concentrations, as well as in a 2-year follow-up.

## 2. Materials and Methods

### 2.1. Participants

In a two-year follow-up study, we involved 79 patients, mean age 18.59 (±3.28), with a diagnosis of mood disorders: Major depressive disorder (MDD) or bipolar disorder (BD). Patients were in-patients or out-patients from the Child and Adolescent Psychiatry Ward, Adult Psychiatry Ward, and Outpatients Clinic at the Department of Psychiatry, Poznan University of Medical Sciences, Poland. A psychiatrist assessed lifetime diagnoses and current mental status examination. The clinical evaluation and biological samples collection were carried out at five visits during a two-year follow-up: the inclusion in the study (baseline visit), then after three, six months, one year, and two years. The diagnosis was verified at each time point by two independent psychiatrists, according to ICD-10 and DSM-IV criteria using a standard instrument to confirm the diagnosis in adolescent patients: The Kiddie Schedule for Affective Disorders—Present and Lifetime Version (KSADS-PL) [21], and in adult patients: Structured Clinical Interview for DSM-IV (SCID) [22]. The severity of the mood symptoms was evaluated with the Hamilton Depression Rating Scale (HDRS-17) [23] and the Young Mania Rating Scale (YMRS) [24]. The cutoff points for depressed mood were HDRS-17 ratings ≥ 8 and for hypomanic/manic YMRS ≥ 12. The Ethics Committee at the Poznan University of Medical Sciences approved the study protocol (permission no. 362/11) in accordance with the Declaration of Helsinki. All study participants were Caucasians of Polish origin from the Greater Poland region. Written informed consent to participate in the study was obtained from the subjects or their legal guardians before the commencement of the study. The exclusion criteria were: any severe medical or neurological illness, intellectual disability disorder, a pervasive developmental disorder, pregnancy. The control group consisted of 31 healthy persons, mean age 21.15 (±2.68). A psychiatrist examined the Control group. Exclusion criteria were: any psychiatric diagnosis, no family history of severe psychiatric disorders, substance abuse, or severe medical problems.

### 2.2. BDNF, proBDNF, EGF, MIF, SCF Serum Levels ELISA Determination

Ten milliliters of venous blood were drawn into anticoagulant-free tubes between 07:30 and 09:30 after overnight fasting. After 1 h incubation, the serum was separated by centrifugation, aliquoted, and stored at −70 until analyses of the entire samples could be achieved. Enzyme-linked immunosorbent assay analyses were performed using BDNF, proBDNF, EGF, MIF, SCF (cat. No.: DY248, DY3175, DY236, DY289, DY255, respectively) DuoSet ELISA Development Kits (R&D System, Minneapolis, MN, USA) according to the manufacturer’s instructions, with minor modifications, as described previously [25]. All samples and standards were run in duplicates. All plates for each protein were run in one batch, on the same kit lot number, by the same experienced operator. To minimize inter-assay variation, all samples derived from one patient were analyzed on the same plate. Proportionally, each plate contained control samples. Intra-assay and inter-assay variability was <5% and <10% CV for each studied protein (accordingly).

### 2.3. Mature BDNF (mBDNF) and mBDNF/proBDNF Ratio Estimation

Additionally, we performed cross-reactivity analysis of BDNF and proBDNF DuoSet ELISA kits. We showed that the Human BDNF DuoSet ELISA kit detects mature BDNF and 100% of the proBDNF (data available on request). Therefore, from the total BDNF concentrations obtained, we subtracted the proBDNF levels. The resulted concentration is mature BDNF (mBDNF). We estimated mBDNF/proBDNF ratio (rBDNF).

### 2.4. Statistical Analyses

The Kolmogorov–Smirnoff test was used to check the normality of the data. All studied proteins concentrations showed non-normal distribution; thus, nonparametric methods were used. The Kruskal–Wallis ANOVA, Mann–Whitney U-test, and paired Wilcoxon signed-rank test was applied in the analyses. Linear regression was used in protein concentrations x symptoms severity analysis. Correction for age and gender was performed using ANCOVA. The significance level was set at *p* < 0.05. The statistical analyses were performed using Statistica v13 software. Power analysis for significant results was performed using the G*Power program [25] and STATISTICA 13.3 (StatSoft, Krakow, Poland).

## 3. Results

Seventy-nine patients were included in the study: 52 with depression, 27 with bipolar disorder in hypomanic/manic (*n* = 17), or mixed (*n* = 10) episode diagnosed according to DSM-IV criteria. At baseline, there were 25 drug-free patients and 54 previously medicated patients treated with: selective serotonin reuptake inhibitors (SSRI), selective noradrenaline reuptake inhibitors (SNRI), tricyclic antidepressants (TCA), neuroleptics (typical or atypical), benzodiazepines or mood stabilizers either in monotherapy (*n* = 28) or polytherapy (*n* = 22). Four patients did not provide reliable information on their psychotropic medications prior to study entry. Depending on the current mood episode, all patients received pharmacological treatment (SSRI or TCA) or mood stabilizers after inclusion in the study. During follow-up visits, 41 patients with depression and 14 patients with BD reached euthymic state at different visits. The overall drop-out rate during the study was 25%. The majority of the reasons was a lack of compliance. We observed significantly higher (*p* = 0.03) drop-out rates from patients with BD baseline diagnosis compared to MDD (44% vs. 21%, respectively). During a two-year observation in the study, 15 patients changed the diagnosis from unipolar to bipolar disorder, and four patients from bipolar II to bipolar I type. There was a significant improvement in HDRS-17 (*p* < 0.000001) or YMRS (*p* < 0.001) scores during treatment in the depression subgroup, as well as in the hypomanic subgroup. The clinical characteristics of the study group are presented in Table 1.

### 3.1. Baseline Comparisons of BDNF, proBDNF, mBDNF, rBDNF, EGF, SCF, and MIF Levels

**Figure 1:**
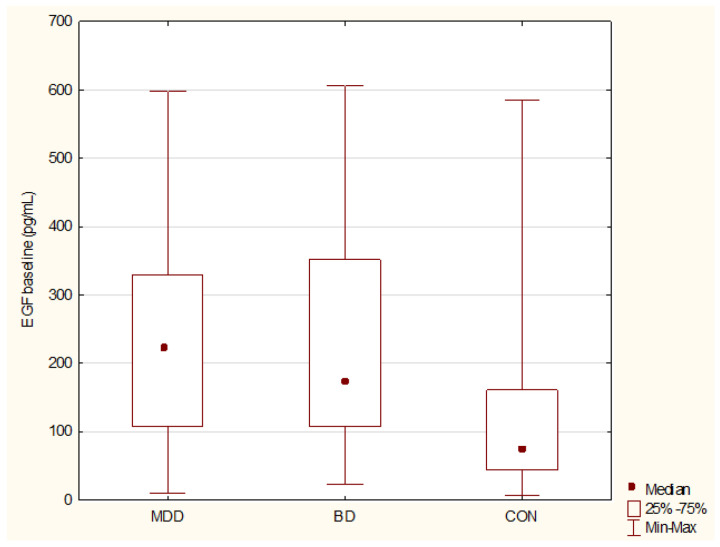
Baseline comparison of epidermal growth factor (EGF) serum levels between major depressive disorder (MDD), bipolar (BD) and controls (CON). BD vs. CON *p* = 0.001; MDD vs. CON *p* = 0.0002. MDD—Major Depressive Disorder; BD—Bipolar Disorder; CON—Healthy Controls.

Comparing baseline serum levels of BDNF, proBDNF, EGF, MIF, and SCF, we detected significant differences between MDD, BD, and CON groups only in EGF levels. Higher EGF was detected in MDD (*p* = 0.0002, power 96%) and in BD group (*p* = 0.001, power 89%) compared to controls. No differences in EGF levels between MDD and BD were detected (*p* = 0.97), Figure 1. Results of Kruskal–Wallis ANOVA comparisons between MDD, BD, and CON at baseline are presented in Table 2.

We did not detect differences in protein levels between MDD and BD patients at baseline (BDNF *p* = 0.42; proBDNF *p* = 0.21; mBDNF *p* = 0.27; rBDNF *p* = 0.37; EGF *p* = 0.98; MIF *p* = 0.79; SCF *p* = 0.61).

Comparing baseline protein levels with regard to diagnosis change to BD in the depressed subgroup during the study, we noticed a statistical trend towards lower EGF levels in the subgroup with diagnosis change (*p* = 0.057). BDNF (*p* = 0.09), proBDNF (*p* = 0.69), mBDNF (*p* = 0.09), rBDNF (*p* = 0.44), MIF (*p* = 0.4), and SCF (*p* = 0.64) concentration did not differ between subgroups of depression without and with diagnosis change.

There were no differences in baseline protein levels in patient subgroups divided with regard to medication status (BDNF *p* = 0.14; proBDNF *p* = 0.28; mBDNF *p* = 0.27; rBDNF *p* = 0.36; EGF *p* = 0.2; MIF *p* = 0.26; SCF *p* = 0.59).

**Figure 2:**
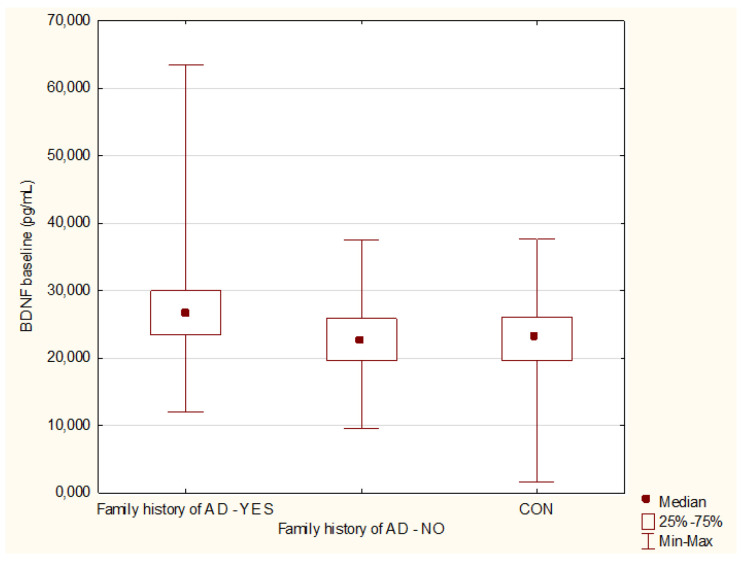
Brain-derived neurotrophic factor (BDNF) levels with regard to family history of affective disorders. Family history of affective disorders: YES vs. No *p* = 0.009. AD—Affective Disorders; CON—Healthy Controls.

Higher BDNF (*p* = 0.009, power 73%) and mBDNF (*p* = 0.03, power 66%) in patients with a family history of affective disorders was detected, Figure 2. No differences were noticed with the rest of the studied proteins with regard to family loading of affective disorders (proBDNF *p* = 0.56; rBDNF *p* = 0.99; EGF *p* = 0.36; MIF *p* = 0.29; SCF *p* = 0.92).

No correlation between baseline symptoms severity scores measured using HDRS-17 and YMRS, and protein levels was discovered in MDD group (BDNF *p*= 0.54; proBDNF *p* = 0.23; mBDNF *p* = 0.31; rBDNF *p* = 0.08; EGF *p* = 0.6; MIF *p* = 0.54; SCF *p* = 0.92) as well as BD group (BDNF *p*= 0.22; proBDNF *p* = 0.12; mBDNF *p* = 0.13; rBDNF *p* = 0.09; EGF *p* = 0.34; MIF *p* = 0.73; SCF *p* = 0.53).

Correction for age and gender revealed a significant effect of age on MIF levels in depressed patients (*p* = 0.01, power 75%), as well as higher BDNF (*p* = 0.03, power 75%) and lower SCF (*p* = 0.004, power 66%) concentrations in depressed females compared to males.

### 3.2. Longitudinal Comparisons of BDNF, proBDNF, mBDNF, rBDNF, EGF, SCF, and MIF Levels

Comparing baseline and euthymic state protein concentrations we found a trend towards an increase in BDNF level in the MDD group during treatment (*p* = 0.06) and a significant decrease in EGF level in the BD group (*p* = 0.02, power 83%). Due to the small size of the study group associated with a high percentage of dropouts, we present the results in all patient groups as a combined group of (MDD + BD) in baseline vs. 24-month visit comparisons. No significant changes during a 2-year follow-up in BDNF, proBDNF, mBDNF, rBDNF, EGF, SCF, and MIF levels were noticed. Results of the Wilcoxon signed-rank test of matched groups are presented in Table 3.

### 3.3. Comparisons of BDNF, proBDNF, mBDNF, rBDNF, EGF, SCF, and MIF Levels—Euthymic State vs. Controls

We detected higher EGF levels in euthymic patients compared to control subjects (*p* = 0.02, power 71%). No differences were detected for (BDNF *p* = 0.17; proBDNF *p* = 0.67; mBDNF *p* = 0.32; rBDNF *p* = 0.85; MIF *p* = 0.26; SCF *p* = 0.55).

## 4. Discussion

To the best of our knowledge, this is the first longitudinal study examining EGF, SCF, and MIF serum concentrations in adolescent and young adult groups with mood disorders. Only several publications concerning BDNF levels in comparable patient groups are available; none have examined mBDNF and proBDNF simultaneously.

The main finding of our study is a disease-dependent significant elevation of EGF in adolescents with mood disorders. Constantly, higher EGF was detected in depressed, hypomanic/manic, and euthymic states compared to healthy controls, making EGF a potential biomarker of mood disorders, especially considering high statistical power. The results of circulating EGF levels studies in mental illnesses are inconclusive. Domenici et al. (2010) reported higher EGF levels in schizophrenia (SCH) but not in major depressive disorder (MDD). These results are especially valuable because of the large (*n* = 741) patient cohort included in the analyses [26]. A significant decrease in EGF plasma levels in MDD was reported by Tian et al. (2012) in a study on a group of 210 patients and matched controls [27], while elevated EGF was found in MDD > 28 years; however, sample size and power of the analysis were low [28]. A comparative study in BD, MDD, and SCH patients (*n* = 40 for each group) was performed by Yamamori et al. (2016) and found an increase in EGF plasma levels in BD compared to healthy controls [29]. EGF levels were measured in the offspring of the bipolar parent in the longitudinal study. Progeny who converted to mood disorder had higher EGF than those with no disease before disease onset, though both groups had lower EGF than healthy controls [30]. Wu et al. (2019) reported no differences in EGF levels in elderly patients diagnosed with MDD [31]. A decrease in EGF levels in recent-onset patients with BD in euthymia was discovered by Bond et al. (2020). Lower EGF correlated with a greater number of past mood episodes and lower bilateral temporal lobe volumes [16]. Microarray expression analysis of MDD patients revealed downregulation of EGF mRNA [32]. Recently published results of post-mortem expression study showed no differences in EGF and BDNF mRNA quantities in a subependymal zone in SCH and BD [33].

In the presented study, increased BDNF and mBDNF in patients with a family history of affective disorders is in line with previous findings reported by Knorr et al. (2017), who found higher levels of BDNF in healthy persons with a family history of depression [34]. Moreover, Duffy et al. (2014) found elevated BDNF levels in the offspring of a bipolar parent [35]. Elevated BDNF levels may act as a possible protective or compensatory mechanism in individuals whose first-degree relatives are diagnosed with mood disorders. Results obtained in different studies concerning the BDNF level in adolescents with mood disorders are inconsistent. Lack of differences in the group of euthymic as well as symptomatic patients with bipolar disorder compared to the control group was detected [36,37,38,39,40]. Correlations with amygdala volume and duration of the medication were reported [37,41] and lack of relationship with hippocampal volume in adolescents with bipolar disorder was found [41,42]. Lower levels of BDNF in adolescents with depression compared to controls was found, with a gender bias with greater reduction in females [43]. Similar results were reported by Pandey et al. (2010) in adult and pediatric depressed patients during a drug-free period, where decreased mRNA BDNF in lymphocytes and protein expression in platelets was found [44]. The most recent study by Lee et al. (2020) on an adolescent MDD group treated with escitalopram found no differences in baseline BDNF level between MDD patients and healthy controls as well as correlations with baseline symptoms severity. No medication effect on BDNF levels was observed [45]. Meta-analyses of BDNF studies in adult patients with MDD [46] or BD [47] confirmed a significant reduction in peripheral BDNF levels in mood disorders. In our research on adolescent patients, we did not notice differences in BDNF levels between depressive or hypomanic/manic episodes compared to the healthy controls.

In our study, baseline MIF levels in depressed or bipolar patients were similar to healthy controls. However, higher MIF concentrations in older subjects in the depressed group were noticed. In the most previously published studies, elevated MIF in patients with MDD was reported. An increase in MIF in first-onset drug-naïve MDD patients [48] and correlation with symptoms severity [49] was detected. A replication study conducted on a large cohort of MDD patients by the same research team replicated previously reported higher MIF levels in MDD, either in a current depressive episode or in the remitted state; no influence of treatment on MIF levels was observed [50]. MIF concentrations were measured in MDD patients who underwent pharmacotherapy using reboxetine or celecoxib. Significantly elevated MIF concentrations at baseline compared to healthy controls and no effect of five weeks of treatment on MIF levels was detected [51]. The impact of two-months lamotrigine and sodium valproate treatment on serum MIF levels was investigated in a group of 140 patients in the depressive episode of BD. No baseline differences were detected compared with healthy controls. A significant decrease in MIF level after treatment was noticed for both drugs used, with a more substantial effect for lamotrigine [52]. Absolute mRNA values of MIF were measured in the two independent samples with MDD diagnosis. Using network analysis, Cattaneo et al. (2016) discovered that MIF interacts with pathways involved in neurogenesis, cell proliferation, and neuroplasticity. The treatment prediction model also established a link with interleukin-1 beta mRNA [53]. No differences in the expression of MIF mRNA in drug-free MDD were found [54]. A genetic study in the adolescent group revealed the association of MIF polymorphisms with anxiety and attenuated cortisol reactivity [55]. Patients from a randomized controlled trial compared mindfulness-based therapy with treatment as usual, with different psychiatric diagnoses: depression, anxiety, or stress and adjustment disorders were included. A post-treatment decrease in MIF levels was reported, but no correlation with improvement in psychiatric symptoms was found [56]. Healthy students were screened with the Beck Depression Inventory and UCLA loneliness scale in the study by Edwards et al. (2010). In the high-depressive symptoms group, MIF levels were 40% higher than in the low depressive symptoms group. Elevated MIF was also associated with a smaller cortisol response to acute stress and lower diurnal morning cortisol values [57].

There are only a few studies concerning SCF measures in psychiatric disorders. In BD patients, decreased SCF levels were detected [48]. Benedetti et al. (2016) reported the effect of combined total sleep deprivation and morning light therapy (TSD + LT) in a group of patients diagnosed with BD in a major depressive episode. Among other proteins studied, SCF plasma levels increased significantly in responders and correlated with gray matter volumes in frontal and parietal cortical areas as well as neural responses in the anterior cingulate and medial prefrontal cortex [20]. Constantly elevated SCF concentrations in the offspring of a bipolar parent in the prodromal phase and after establishing MDD diagnosis was reported [30]. Lower methylation of the gene encoding MIF in BD was detected [58]. Our results did not support the role of MIF in adolescent mood disorders.

## 5. Conclusions

The main finding of our study is a disease- not state-dependent significantly elevated EGF level in adolescent patients with mood disorders compared to healthy controls. Pharmacotherapy did not influence serum concentrations of the studied proteins, except for a decrease in EGF levels between baseline and euthymic state. However, even in euthymia, we detected higher EGF in patients compared to the controls. This finding suggests that EGF might be a potential biomarker of BD. Our results on BDNF/proBDNF are inconsistent with the most published studies indicating a decrease in BDNF in MDD and BD. However, an elevated level of BDNF was detected in patients with a family history of affective disorders. We did not notice any differences in MIF and SCF levels in our group, nor correlations of all studied proteins with symptom severity. Numerous studies have tried to find reliable peripheral biomarkers in psychiatric disorders during the last decade, but the results are inconsistent or even conflicting. Replication studies on large, homogenous cohorts of patients are necessary. As mental illnesses are diseases of very complex etiology, a panel of biomarkers rather than single proteins is expected to be of clinical value.

Limitations of this study include a relatively small sample size, high drop-out rate, and a lack of an exact age-matched control group. Future studies with larger samples and a more extended period of longitudinal observation are necessary.

## Figures and Tables

**Table 1:** Characteristics of the study group at baseline.

	All Patients	MDD	BD	Control
*n*	79	52	27	31
Female/Male	56/23	39/13	17/10	26/5
Mean age	18.59 (±3.28)	18.67 (±3.54)	18.44 (±2.79)	21.1 (±2.68)
Mean age at illness onset	16.77 (±2.76)	16.82 (±2.96)	16.6 (±2.31)	NA
Drug free yes/no	25/54	21/31	4/23	
Inpatient/outpatient	60/19	36/16	24/3	
Mean number of hospitalization	1.27 (±0.74)	1.25 (±0.80)	1.29 (±0.62)	
Family history of bipolar disorder = yes/no	38/41	29/23	9/18	
Change of diagnosis to BD	19	15	4	
HDRS-17	14.62 (±8.27)	19.37 (±5.30)	5.48 (±4.27)	
YMRS	6.71 (±9.10)	1.04 (±1.55)	17.6 (±7.47)	
BDNF (pg/mL) (mean ± SD)	25,154.48 (±7937.7)	24,118.76 (±6318.7)	27,149.21 (±10219.1)	22,349.56 (±7078.5)
proBDNF (pg/mL) (mean ± SD)	2202.25 (±2187)	2379.82 (±2282.2)	1860.26 (±1987)	3224.38 (±3336.3)
mBDNF (pg/mL) (mean ± SD)	22,952.23 (±8201.1)	21,738.93 (±6474.5)	25,288.95 (±10532.5)	19,125.18 (±7879.6)
rBDNF (mean ± SD)	23.97 (±24.4)	23.57 (±25.8)	24.79 (±21.52)	19.09 (±18.4)
EGF (pg/mL) (mean ± SD)	229.95 (±154.1)	230.77 (±154.9)	228.37 (±155.3)	156.05 (±165.1)
MIF (pg/mL) (mean ± SD)	1871.55 (±1035.4)	1927.63 (±1136.4)	1763.55 (±815.28)	2295.74 (±1471.7)
SCF (pg/mL) (mean ± SD)	155.02 (±100.1)	161.49 (±116.2)	142.86 (±59.31)	168.58 (±150.8)

MDD—Major Depressive Disorder; BD—Bipolar Disorder; HDRS-17-Hamilton Depression Rating Scale (17-item); YMRS—Young Mania Rating Scale; BDNF—Brain-Derived Neurotrophic Factor; proBDNF—precursor BDNF; mBDNF—mature BDNF; rBDNF—BDNF/proBDNF ratio; EGF—Epidermal Growth Factor; MIF—Migration Inhibitory Factor; SCF—Stem Cell Factor.

**Table 2:** Baseline comparisons of protein levels between major depressive disorder (MDD), bipolar disorder (BD) and healthy controls (CON).

	Mean Rank					
	MDD	BD	CON	H	Χ^2^	*p*
BDNF	56.06	62.22	48.71	2.62	1.22	0.2699
proBDNF	58.14	49.15	56.61	1.46	1.65	0.4812
mBDNF	55.33	63.56	48.77	3.10	1.73	0.2121
rBDNF	52.98	60.28	52.39	1.12	3.22	0.5727
EGF	63.67	63.74	37.00	15.67	10.84	0.0004
MIF	56.02	54.59	57.16	0.09	0.61	0.9546
SCF	52.85	49.02	49.71	0.36	0.58	0.836

Kruskal–Wallis ANOVA; MDD—major depressive disorder; BD—bipolar disorder; CON—healthy controls; BDNF—brain-derived neurotrophic factor; proBDNF—precursor BDNF; mBDNF—mature BDNF; rBDNF—BDNF/proBDNF ratio; EGF—epidermal growth factor; MIF—migration inhibitory factor; SCF—stem cell factor.

**Table 3:** Longitudinal comparisons of brain-derived neurotrophic factor (BDNF), precursor BDNF (proBDNF), mature BDNF (mBDNF), BDNF/proBDNF ratio (rBDNF), epidermal growth factor (EGF), migration inhibitory factor (MIF), and stem cell factor (SCF) levels.

	Baseline vs. Euthymia				Baseline vs. 24 Month	
	MDD		BD		MDD + BD	
	Z	*p*	Z	*p*	Z	*p*
BDNF	1.91	0.06	1.57	0.12	0.96	0.34
proBDNF	1.02	0.31	1.15	0.25	0.05	0.96
mBDNF	1.33	0.18	1.08	0.28	0.795	0.43
rBDNF	0.33	0.74	0.31	0.75	0.435	0.66
EGF	1.04	0.30	2.41	0.02	0.665	0.51
MIF	0.51	0.61	0.10	0.92	0.185	0.85
SCF	0.28	0.78	0.18	0.86	1.495	0.14

Wilcoxon signed-rank test; MDD—Major Depressive Disorder; BD—Bipolar Disorder; BDNF—Brain-Derived Neurotrophic Factor; proBDNF—precursor BDNF; mBDNF—mature BDNF; rBDNF—BDNF/proBDNF ratio; EGF—Epidermal Growth Factor; MIF—Migration Inhibitory Factor; SCF—Stem Cell Factor.

## Data Availability

Data are available on request.
